# Retrograde Endopyelotomy with Cutting Balloon™ for Treatment of Ureteropelvic Junction Obstruction in Infants

**DOI:** 10.3389/fped.2016.00072

**Published:** 2016-07-08

**Authors:** Alberto Parente, Laura Perez-Egido, Rosa Maria Romero, Ruben Ortiz, Laura Burgos, Jose Maria Angulo

**Affiliations:** ^1^Paediatric Urology, Hospital General Universitario Gregorio Marañón, Madrid, Spain

**Keywords:** hydronephrosis, endopyelotomy, cutting, balloon dilation, infant

## Abstract

**Purpose:**

The aim of this study is to analyze results of retrograde endopyelotomy with cutting balloon for treatment of ureteropelvic junction obstruction (UPJO) in infants.

**Methods:**

We routinely treat patients with UPJO under 18 months of age with retrograde high-pressure balloon dilatation of the pelviureteric junction (PUJ). During the procedure, in these cases where narrowing at the PUJ persists, endopyelotomy with cutting balloon is performed. Endopyelotomy is performed over guidewire with 5-mm Cutting Balloon™ under fluoroscopic control. Double-J stents is left *in situ* for 4 weeks. We retrospectively analyzed the postoperative, clinical, and radiological outcome infants treated with cutting balloon endopyelotomy between 2007 and 2015.

**Results:**

Sixteen patients required cutting balloon endopyelotomy to achieve complete resolution of narrowing of the waist observed during high-pressure balloon dilatation of the PUJ. Mean operative time was 35 ± 21 min (mean ± SD) and hospital stay was <24 h in all patients. Complete resolution of the narrowing at the PUJ under fluoroscopy was achieved in all cases, with no perioperative complications. One patient presented with urinary tract infection, postoperatively (Clavien grade II). Preoperatively, all cases had grade IV SFU hydronephrosis with parenchymal thinning. During follow-up, resolution of the hydronephrosis was observed in 11 patients (grade I SFU). In four infants, there was an improvement of the hydronephrosis (grade II SFU) and the renogram curve. In one case, an open pyeloplasty was required due to persistent hydronephrosis and obstructive curve.

**Conclusion:**

We believe that endopyelotomy with cutting balloon could be a valid and safe option in minimally invasive management of UPJO in infants.

## Introduction

The reference standard for treatment of ureteropelvic junction obstruction (UPJO) has been open pyeloplasty, with a success rate of over 90% (1). Open surgery, however, is associated with significant morbidity, including the need of a large incision, longer convalescence, and increased postoperative pain.

In the last decade, minimally invasive approach to UPJO has expanded aiming to reduce the morbidity associated with open surgery and with the goal to achieve comparable outcomes. However, evidence is still lacking to demonstrate similar outcomes to classical open pyeloplasty (2, 3).

Endopyelotomy is aimed to produce full-thickness longitudinal incision of the narrow segment at the pelviureteric junction (PUJ), followed by stenting and drainage to allow regeneration of a PUJ of adequate diameter around the stent. Endopyelotomy has been reported with an antegrade and lately with a retrograde approach in children, with different devices and outcomes. One of the main limitations of this technique has been the lack of an adequate instrumentation that can successfully allow an endopyelotomy in small children (4, 5).

Recently, a new system for balloon angioplasty was introduced. The peripheral cutting balloon microsurgical dilatation device (PCBD) combines the features of conventional balloon angioplasty with advanced microsurgical capabilities (Boston Scientific, Natick, MA, USA). This device was originally designed for in-stent treatment for coronary artery stenosis and has been expanded to additionally treat fibrotic vascular stenosis, including vein bypasses and dialysis–fistulae–stenosis, with potential for a better dilatation of ischemic and fibrotic lesions resistant to conventional percutaneous transluminal angioplasty (6, 7).

Cutting Balloon™ has already been used successfully in the urinary tract stricture, specifically in the treatment of primary obstructive megaureter (8, 9).

The aim of this study is to analyze the results of retrograde endopyelotomy with cutting balloon for treatment of UPJO in infants. The proposed new use for this device was requested to the hospital committee board for innovation, and approval was obtained.

## Materials and Methods

We review retrospectively the case notes of our series of patients <18 months of age with UPJO treated with retrograde endopyelotomy using cutting balloon between 2007 and 2015 (*n* = 16). No patient needed bilateral treatment.

As per our unit protocol, infants (<18 months) with UPJ obstruction requiring surgical treatment, retrograde dilation of the PUJ with high-pressure balloon is the first-line treatment. This is carried out after performing retrograde pyelography, dilating UPJO with 5- or 6-mm high-pressure balloon under fluoroscopic control. If we observed too much difficulty to pass the contrast into the renal pelvis and the “waist” in the balloon was persistent after 20 s, the technique was modified to a retrograde endopyelotomy with cutting balloon.

### Technique Description

Cystoscopy is performed with the patient in lithotomy position using a 9.5-Fr cystoscope with a 5-Fr instrumentation channel. At the start of the procedure a 4-Fr ureteral catheter is inserted to perform a retrograde pyelography. Then, a 0.014″-guidewire is negotiated up to the renal pelvis. If it was not possible, a 0.035″-hydrophilic guidewire was used (Radiofocus Guide Wire; Terumo Corp., Somerset, NJ, USA), because it goes in easier in the retrograde direction inside the ureteral lumen.

**Figure 1:**
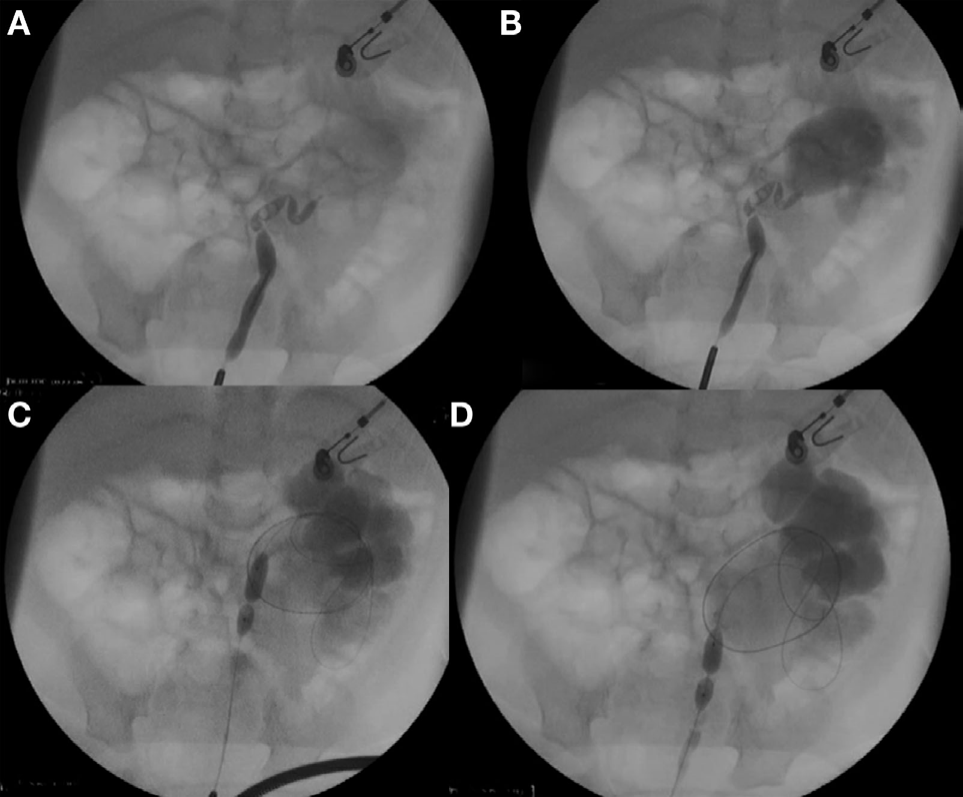
**(A,B)** Retrograde pyelography with extremely difficult progression of the contrast in the renal pelvis. **(C,D)** High-pressure balloon at 15 atm cannot overcome the “waist” within 20 s.

**Figure 2:**
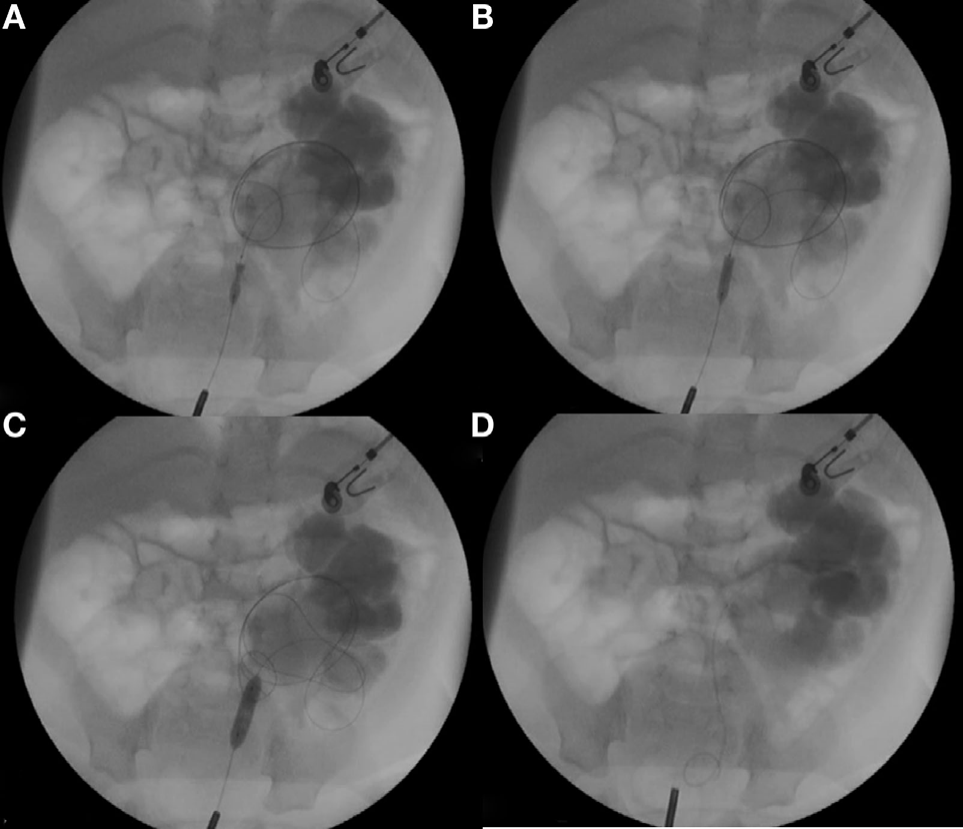
**(A,B)** Cutting Balloon™ was inflated at the level of ureteropelvic junction to up to 12 atm. **(C)** Dilatation was then completed with the 6 mm balloon dilatation previously used. **(D)** Double-J was placed in the renal pelvis.

Dilatation of UPJO with a 6-mm semi-compliant high-pressure balloon (Rx Muso Terumo Corp.) inserted over the guidewire is done in all cases with fluoroscopic control. When progression of the contrast progression in the renal pelvis was extremely difficult and resolution of the “waist” or narrowing at the PUJ is not achieved within 20 s (high-pressure balloon inflated at 15 atm), an endopyelotomy with cutting balloon (Figure 1) was subsequently performed. For this purpose, a 3-, 5- or 4-mm diameter Cutting Balloon™ is inflated at the level of ureteropelvic junction to up to 12 atm. Dilatation was then completed with the 6-mm balloon dilatation previously used (Figure 2).

The bladder catheter is removed 16–18 h after the procedure, and the patient is discharged home on oral antibiotic prophylaxis. The double-J stent is removed as an outpatient procedure 4 weeks, postoperatively.

In order to minimize ionizing radiation exposure, operators reduce the effective dose to themselves, the patient, and other personnel by minimizing the use of digital subtraction acquisitions, avoiding lateral angulations, using higher magnification levels when possible, and being diligent about the use of shielding during fluoroscopy cases. We routinely use protection of gonads during the visualization of the UPJO. In the past, we carried out an institutional audit on radiation exposure to pediatric patients having UPJO endoscopic dilatation (10).

### Postoperative Control

Patients are followed as per our unit protocol with outpatient clinical review and radiological follow-up consisting of renal ultrasonography at 3, 6 and 12 months, postoperatively. Diuretic renography is performed at 3 months, postoperatively.

For the study, preoperative data are collected from clinical case notes, including degree of hydronephrosis (SFU), measurement of the renal parenchymal thickness, and indication for surgical treatment. We analyze operative outcome and postoperative clinical and radiological outcome. Statistical analysis is done using paired *t*-test, *p* values <0.05 were considered statically significant. Statistical analysis is performed using SPSS statistical software.

## Results

Between June 2007 and March 2015, 77 children <18 months of age were treated with high-pressure balloon dilatation of UPJO. Sixteen of them (21%) require endopyelotomy with Cutting Balloon, which make up our study group (10 boys, 6 girls). Patients’ mean age at surgery was 7.8 ± 5.5 months (range 2–17 months) and mean postsurgical follow-up time was 61 ± 27 months. The right kidney was affected in 6 patients and left kidney in 10.

Seventy percent of patients had an antenatal diagnosis of hydronephrosis. Diagnosis of PUJO was based on serial renal ultrasound scan [Society for Fetal Urology grade, anteroposterior diameter, and parenchymal thickness (11)] and diuretic renogram MAG-III (12). In all cases, a micturating cystourethrogram was done before the endourological procedure.

Indication for surgery was a grade IV hydronephrosis with obstructive diuretic drainage curve (all cases) and differential renal function ≤40% (seven patients), worsening hydronephrosis with parenchymal thinning and obstructive curve renogram (six patients), or recurrent febrile infections (three patients) (13).

Mean operative time was 35 ± 21 min. There were no intraoperative complications. Extravasation of contrast was noted in seven cases, but this is not considered as a complication, as is not being described as associated with poor outcome. All patients had a 24-h hospital admission, and no oral painkillers were required after discharge.

One postoperative complication was recorded, consisting of an urinary tract infection (complication Clavien grade II). Re-admission and early removal of the double-J stent was required in this patient [complication grade II (14)]. Hematuria disappeared in all patients in <12 h, postoperatively.

The double-J stent was removed after a mean time of 31 ± 8 days, as an outpatient procedure, with no intraoperative or postoperative complications.

Preoperative ultrasound showed hydronephrosis grade IV in all infants with mean anterior–posterior diameter of the renal pelvis of 30 ± 11 mm. The mean parenchymal thinning was 3.9 ± 1.0 mm. Seven infants had decreased or impaired renal function (<40%), according to the renogram. The preoperative curve pattern was obstructive in all patients.

During follow-up, renal ultrasound showed improved of hydronephrosis grade according to SFU in 15 patients (postoperative SFU grade: 11 patients grade I and 4 patients grade II). The analysis of the anterior–posterior diameter of the renal pelvis pre and postoperatively decreased from 30 ± 11 to 12 ± 5 mm (*p* < 0.01) at 12-month postoperative renal scan. The mean parenchymal thinning increased from 3.9 ± 1.0 to 8.7 ± 2.8 mm at 12-month postoperative follow-up (*p* < 0.01).

The preoperative renogram curve was obstructive in all patients. This was completely normalized in nine patients and with significant improvement in six patients. In one patient, postoperative renogram was obstructive, without improvement in the ultrasound degree of hydronephrosis and finally required an open pyeloplasty (Table 1).

**Table 1:** **Patient outcomes**.

Patient	Age (months)	Preoperative APD renal pelvis	Postoperative APD renal pelvis	Preoperative parenchyma	Postoperative parenchyma	Preoperative differential renal function	Postoperative differential renal function	Preoperative renogram curve	Postoperative renogram curve	Outcome
				Thickness (mm)	Thickness					
1	17	19	15	6	10	50	50	Obstructive	Semiobstructive	Successful
2	11	20	18	5	6	45	47	Obstructive	Obstructive	Re-pyeloplasty
3	17	22	13	5	9	45	48	Obstructive	Normal	Successful
4	4	40	6	2	15	56	49	Obstructive	Semiobstructive	Successful
5	5	32	10	3	6	39	45	Obstructive	Normal	Successful
6	3	60	6	2.5	5	39	42	Obstructive	Semiobstructive	Successful
7	2	43	20	3	10	24	35	Obstructive	Normal	Successful
8	5	20	13	5	10	20	35	Obstructive	Semiobstructive	Successful
9	13	28	14	4	9	43	48	Obstructive	Normal	Successful
10	6	37	17	4	6	44	47	Obstructive	Semiobstructive	Successful
11	9	20	14	4	8	55	47	Obstructive	Normal	Successful
12	18	40	12	5	10	31	32	Obstructive	Normal	Successful
13	3	30	2	3	10	39	48	Obstructive	Normal	Successful
14	4	23	15	4	7	45	46	Obstructive	Semiobstructive	Successful
15	3	28	8	4	8	49	48	Obstructive	Normal	Successful
16	3	19	13	4	7	38	41	Obstructive	Normal	Successful

*APD, anterior–posterior diameter*.

## Discussion

Open pyeloplasty is considered the gold standard for the treatment of UPJO. In the last two decades, modern endourological instruments and techniques have shifted toward the management of UPJO using minimally invasive procedures (2). All of these techniques share the advantages of short operative time, minimal morbidity, decreased postoperative analgesic requirements, shorter hospitalization, and early recovery.

Cutting balloon is a special balloon with four microsurgical blades that are activated when the balloon is inflated. They expand radially and deliver longitudinal incisions, relieving the hoop stress of UPJO. These blades do not cut deeply, avoiding the full section of the vessel or ureter. This is demonstrated by the low number of complications described when used in angiology (15). Thus, it allows us to perform a first section of the fibrotic area safely, for subsequent completion of dilatation with the balloon previously used and getting an adequate passage of urine at the UPJ.

Although many patients present contrast extravasation, this also occurs in endopyelotomy with laser or cold-knife (16) and in high-pressure balloon dilatación of UPJO (17). We do not consider extravasation to be a complication, because it has been shown not to have a negative impact on postoperative complications or outcomes.

Ureteroplasty with a cutting balloon is a recognized treatment option for ureteric strictures in adults (18, 19). Although there are several studies analyzing the use of such devices in the literature, few, until recently, had analyzed the use of the PCBD. In these studies, the peripheral Cutting Balloon device is a well tolerated, rapid, safe, and reliable method for treating ureteric strictures, associated with low morbidity and short hospital stay (20).

Several studies, however, have analyzed the use of the Acucise device (Applied Medical Resources), designed specifically for the treatment of UPJ and ureteric obstructions. This device was first described for treating ureteric strictures, in 1993 (21). In the largest study, a multicentric trial analyzing both endopyelotomies and endoureterotomies, the authors concluded that ureteroplasty with several cutting balloon devices like Acucise is effective in the majority of cases, producing patency rates mirroring other endourological procedures, with the advantage of being cheaper and quicker (22). We believe that the peripheral Cutting Balloon is safer, as reflected in our series.

The peripheral Cutting Balloon has been rarely used in children, and only few studies analyze its use in congenital stenosis of the urinary tract. Indeed, it has only been reported in the treatment of primary obstructive megaureter (8, 9), with very good results and minimal complications. To the best of our knowledge, this is the first publication about Cutting Balloon™ in UPJO, in infants.

We believe that results are acceptable, with a success rate of 94%. Complications were rare, and time of hospital stay is much lower than usual in open or laparoscopic pyeloplasty. In our current practice, we do not use the cutting balloon routinely due to the high cost of this device and the good results obtained with high-pressure balloon dilation (17). Additionally, the peripheral Cutting Balloon has a profile that makes it difficult to use through the instrumentation channel of 9.5-Fr cystoscope, so it is necessary to use with no direct vision, only with fluoroscopic control. However, it is possible that the good results of endopyelotomy with Cutting Balloon lead us to reconsider this decision to enhance our success rate with high-pressure balloon dilation alone (84% in our experience).

Some authors prefer an anterograde approach for endoscopic dilatation or endopyelotomy under 18 months of age (23). In our opinion, percutaneous approach is not free of complications. We believe that the use of cutting balloons for retrograde endopyelotomy allows us to minimize damage to the kidney and reduce complications and operative time, obtaining comparable results.

Operative time and hospitalization stay in our study is very low, compared with a recent series of open or laparoscopic pyeloplasty in infants (24, 25). We believe this is one of the major advantages of this technique, in addition to the absence of scars or sequelae.

When comparing our results with other endopyelotomy, we found that most groups perform better results in secondary UPJO than in primary UPJO. While outcomes success in primary UPJO achieves 60–70% (23, 26), Kim et al. achieves 94% success rate in secondary (23). Our results are good in primary UPJO. Furthermore, our results are consistent with the literature describing few intra or postoperative complications.

Of course, the radiation exposure of children is an issue that concerns us. So, operators reduce the effective dose to themselves, the patient, and other personnel by minimizing the use of digital subtraction acquisitions, avoiding lateral angulation, using higher magnification levels when possible, and being diligent about the use of shielding during fluoroscopy cases. Although stochastic risks were small, it is highly recommended to employ all the available methods and techniques developed for patient radiological protection. A strong coordination between team members is advisable for improving the dose optimization (10).

Among the limitations of the study, we find that it is a retrospective study with no control group. Furthermore, a larger number of patients could exclude the existence of rare but potentially serious complications, which we did not encounter in our experience. A more extensive experience could possibly help to define criteria for the use of cutting balloon compared to high-pressure balloon.

We believe that endopyelotomy with cutting balloon could be a valid and safe option. It could be an alternative in the minimally invasive treatment of UPJO in infants, mainly in these cases that show incomplete resolution of the narrowing or waist during endoscopic dilatation.

## Ethics Statement

This study was carried out in accordance with the recommendations of “Comite Etico del Hospital Gregorio Marañon.” All subjects gave written-informed consent in accordance with the Declaration of Helsinki.

## Author Contributions

The above listed authors (AP, LP-E, RO, LB, RR, and JA) were involved in the design of the submitted study. They were involved in the acquisition, analysis, and interpretation of the data. Each author was involved in drafting the manuscript and editing it for accuracy and content. Each author was included in approving the manuscript for final approval before submission. Additionally, all authors agreed to be responsible for all aspects of the submitted work.

## Conflict of Interest Statement

The authors declare that the research was conducted in the absence of any commercial or financial relationships that could be construed as a potential conflict of interest.
